# NASA EOSDIS Data Identifiers: Approach and System

**DOI:** 10.5334/dsj-2017-015

**Published:** 2017-04-04

**Authors:** Lalit Wanchoo, Nathan James, Hampapuram K. Ramapriyan

**Affiliations:** 1 ADNET Systems, Inc., Lanham, MD 20706, US; 2 Earth Science Data and Information System Project (Code 423), NASA Goddard Space Flight Center, Greenbelt, MD 20771, US; 3 Science Systems and Applications, Inc., Lanham MD 20706, US

**Keywords:** DOI, Identifier, ESDIS, NASA, EOSDIS, EZID, Metadata

## Abstract

NASA’s Earth Science Data and Information System (ESDIS) Project began investigating the use of Digital Object Identifiers (DOIs) in 2010 with the goal of assigning DOIs to various data products. These Earth science research data products produced using Earth observations and models are archived and distributed by twelve Distributed Active Archive Centers (DAACs) located across the United States. Each data center serves a different Earth science discipline user community and, accordingly, has a unique approach and process for generating and archiving a variety of data products. These varied approaches present a challenge for developing a DOI solution. To address this challenge, the ESDIS Project has developed processes, guidelines, and several models for creating and assigning DOIs. Initially the DOI assignment and registration process was started as a prototype but now it is fully operational. In February 2012, the ESDIS Project started using the California Digital Library (CDL) EZID for registering DOIs. The DOI assignments were initially labor-intensive. The system is now automated, and the assignments are progressing rapidly. As of February 28, 2017, over 50% of the data products at the DAACs had been assigned DOIs. Citations using the DOIs increased from about 100 to over 370 between 2015 and 2016.

## Introduction

Since 1994, NASA’s Earth Observing System (EOS) Data and Information System (EOSDIS) has carried out its charter to manage, archive, and distribute data products derived from Earth observing instrument data and environmental models. Currently, over 10,000 unique research data products are being archived and distributed by twelve Distributed Active Archive Centers (DAACs) located across the United States. Each DAAC serves a user community with a different Earth science discipline orientation and, accordingly, provides unique services and tools for discovering and accessing data tailored to meet the needs of that particular community. User requirements include not only convenient access to data but also to the supporting information needed for publishing the research results from the scientific use of the data.

The Earth Science Data and Information System (ESDIS) Project is responsible for managing the 12 EOSDIS DAACs (Murphy and Ramapriyan 2016). Though each DAAC has a different discipline focus, they all have the same types of data management functions: ingest, archive, distribution, and documentation. Since data are a major part of scientific research, proper citation of the data is becoming a significant requirement for research publications (COPDESS 2015). Therefore, the ESDIS Project requires each DAAC to prominently publicize its citation policy via the web and communications that accompany the data. Data citation acknowledges the author’s sources, promotes the reproduction of research results, and makes it easier to identify and find the data (ESIP 2012; DCSG 2014). Creating a data citation clearly benefits both the scientific community and the data center. In addition, by providing proper data citations, the data products become more easily discoverable. In turn, it is likely that there will be an increase in data distribution resulting in increased scientific output. EOSDIS’s 12 DAACs have important data and need to be cited in the scientific publications. This requires a citation policy and persistent identifiers that can be used in citations.

Non-availability of the data could leave users unable to access the data or the information about the data that they have used in their research. This creates a situation wherein scientists may not be able to perform verification and replication of the results of their study and analysis. Recognizing the benefits of data citations, the ESDIS Project evaluated the various approaches for assigning permanent identifiers to data products stored at the DAACs. After considering several options analyzed by Duerr et al. (2011), the ESDIS Project agreed on the use of Digital Object Identifiers (DOIs) for permanently identifying its data collections.

In 2012, the ESDIS Project staff implemented a process for assigning and registering DOIs for data products archived, and distributed by the EOSDIS data centers. It is also to be noted that two of the 12 DAACs – Oak Ridge National Laboratory (ORNL) DAAC and the Socio-Economic Data and Applications Center (SEDAC) – had been using dataset DOIs before their broader usage through the ESDIS Project by the other DAACs. More recently, the ESDIS Project has developed a largely automated DOI registration system based on inputs from the Earth Science Data System Working Groups and the DAACs regarding the DOI assignment process (Wanchoo and James 2014). In addition, the ESDIS Project has developed guidelines for the complete process of registering and maintaining the identifiers, a list of applicable models for creating and assigning DOIs, and guidelines for the landing pages. To support the registration of the identifiers, in February 2012, the ESDIS Project started using the California Digital Library (CDL) EZID system for registering EOSDIS-related DOIs. Since that time the EOSDIS DAACs have submitted over 4000 requests for registering DOIs for their datasets. To facilitate DOI assignment and registration, the ESDIS Project has developed a largely automated system with special emphasis on (a) reviewing DOI metadata, (b) assigning DOI names if requested, and (c) reserving, registering, and updating the DOIs. One of the key features of the system is to give data providers the flexibility of reserving the DOI, which allows data providers to embed and test the DOI in the data product metadata before formally registering the DOI with EZID. In addition, the DOI update process allows the changing of any DOI metadata (except the DOI name unless the name has not yet been registered).

## Assigning a DOI Name

A DOI is permanent. When the DOI is assigned and registered, it can always be used to locate the data object to which it refers. One of the key values of defining a permanent, unique identifier is that even if the Internet location (URL) of the object changes, since the DOI stays the same, the user can always find the data by searching for the DOI as long as the DOI metadata are updated with the new URL. This means that an archive can change the URL of a data product without affecting the validity of previous references in already published literature (Duerr et al 2011; Wanchoo and James 2014). In addition, the publisher (or archive-distributor) of the object can change, while still supporting the same DOI as the object’s permanent locator by the new publisher.

The structure of a DOI is as follows:

resolving authority:[prefix]/[suffix]

The resolving authority redirects the specified DOI to the landing page developed by the data provider. The resolving authority for ESDIS Project DOIs is dx.doi.org.

In the structure displayed above, the prefix consists of a number assigned by the DOI service provider to each DOI registering agency. The ESDIS Project prefix assigned by EZID is 10.5067 and will always be the same for all DOIs that are registered by the ESDIS Project with EZID. The forward slash character (“/”) is used as a delimiter between prefix and suffix. The suffix is a string developed by the data provider assigning the DOI identifier and has no significant restrictions on the format except that it should be unique. The recommendation, however, is to keep it simple and short for ease of use. DOIs are case-insensitive; therefore, letters coded in upper or lower case will refer to the same DOI. Once a DOI is registered it can never be changed or deleted.

### Suggested DOI Suffix Models

To provide guidelines for the suffix, the ESDIS Project has suggested the following models for the data provider’s consideration:

- [mission]/[instrument]/data[m][n]
- [campaign]/[platform group]/data[m][n]
- [program]/[measurement group]/data[n]
- [measurement group]/data[n]
- [instrument]/[shortname.version]
- Opaque ID

where [m] depicts the processing level of the product, and [n] depicts a sequence number that is assigned on a first-come-first-served basis. New product versions receive new DOIs and are assigned a new sequence number.

A few examples include:

- Aura HIRDLS level 2 data product
  - 10.5067/Aura/HIRDLS/DATA201
- MEaSUREs GSSTF Level 3 data product
  - 10.5067/MEASURES/GSSTF/DATA301
- Aqua AMSR-E data product
  - 10.5067/AMSR–E/AE_OCEAN.003
- Operation IceBridge data product
  - 10.5067/IGNSHKO4RRUC (opaque string)

Note that where the DOIs are structured, having semantic meaning, it is expected that the meaning will not change over time. For example, it is not expected that platform and instrument names will change. Even in the unlikely event of their changing, the DOIs will remain as they were originally assigned. Any such changes would be reflected in the associated metadata records.

The following paragraphs briefly describe the process to assign, reserve and register DOIs.

#### Assign DOIs

DOIs are assigned to NASA Earth science data products by request of the DAAC responsible for the archive and distribution of those products. To request a DOI assignment, the DAAC (data provider) must provide a minimal set of descriptive information for each data product requiring a DOI. In turn, the ESDIS Project creates a unique (structured or opaque) DOI, assigns it to the data product, and maintains that DOI assignment in a database.

#### Reserve DOIs

DOIs that are assigned to NASA data products but are not ready for registration with EZID can be reserved. This feature allows DAACs to request DOIs for data products that are not yet publicly available and for the ESDIS Project to ensure DOI names are unique and are not assigned to any other products held by other DAACs before registration. Reserved DOIs can be changed or deleted before they have been registered.

#### Register DOIs

To make DOIs accessible to the public, they must be registered with the EZID service. The ESDIS Project submits DOIs for EZID registration after ensuring that 1) the data provider has provided all the mandatory data product metadata required by EZID, 2) the DOI has a resolvable link to the product’s active landing page, and 3) the product is being actively distributed to the public. Once registered, the DOIs cannot be changed or deleted.

## Roles and Responsibilities

There are three organizations, EZID, DAACs, and the ESDIS Project that are involved in the ESDIS DOI system (Moses and Behnke 2012; Wanchoo and James 2014). Each has following unique responsibilities:

- EZID is the service provider that provides the ESDIS Project access to the DOI handling system (24 hours a day and 7 days a week) to register and resolve DOIs for ESDIS-registered DOIs.
- The DAACs are the content providers and are responsible for providing the ESDIS Project with the data product metadata as shown in Table 1, developing and maintaining the data product-related landing page, and assisting the project in DOI maintenance by providing any changes in the product metadata or landing page URL.
- The ESDIS Project is the registration service provider and is responsible for managing the uniqueness of the DOIs when creating them. In addition, the project provides guidance to the data provider in developing a DOI identifier suffix, reviewing the DOI name and metadata for its completeness, assigning DOIs to the products if requested, and reserving, registering, as well as updating DOIs. The ESDIS Project maintains a wiki website that lists DOIs with the product metadata that have been reserved and/or registered.

## DOI Metadata Requirements

Metadata requirements are primarily driven by the DOI data model described in the DataCite metadata documentation and DataCite has listed 18 properties (attributes) for registering a DOI with only six being mandatory (DataCite Metadata Working Group 2016). The ESDIS Project has reviewed these requirements and chosen to use only the mandatory attributes for DOI registration. These attributes define a data product for the accurate and consistent identification of data for citation. The URL attribute is mandatory for the ESDIS DOI model because it provides the location of the data product’s landing page, which describes and provides the link to access the data product. The ESDIS Project has added additional optional metadata requirements for keeping track of DOIs in relationship to the data products that are being distributed by EOSDIS and also will be helpful in tracking DOI referencing in the literature. These attributes are listed in the Table 1.

## DOIs in Product Metadata

The ESDIS Project encourages the Data Providers to embed the DOI information in the science data product metadata. This will make the DOI easy to find when users wish to cite the data product. At a minimum, the product metadata file must include two attributes related to the DOI:

1. The DOI resolving authority: this attribute would define the authoritative service for use with DOI values in resolving to the URL location and may include:
  - The authority attribute name: identifier_product_doi_authority
  - The authority attribute value: http://dx.doi.org/
2. The DOI name: the DOI values can be set in both the “collection-level” and “granule-level” metadata for the same data product. Note that a collection is defined as a set of files (or granules) representing a given digital data product. All of the granules for one product will have the same DOI value. These attributes may include:
3. The DOI attribute name: identifier_product_doi
  - The DOI attribute value: DOI of the product, e.g., 10.5067/MODIS/MOD17A3H.006

## DOI Processing Workflow

**Figure 1:**
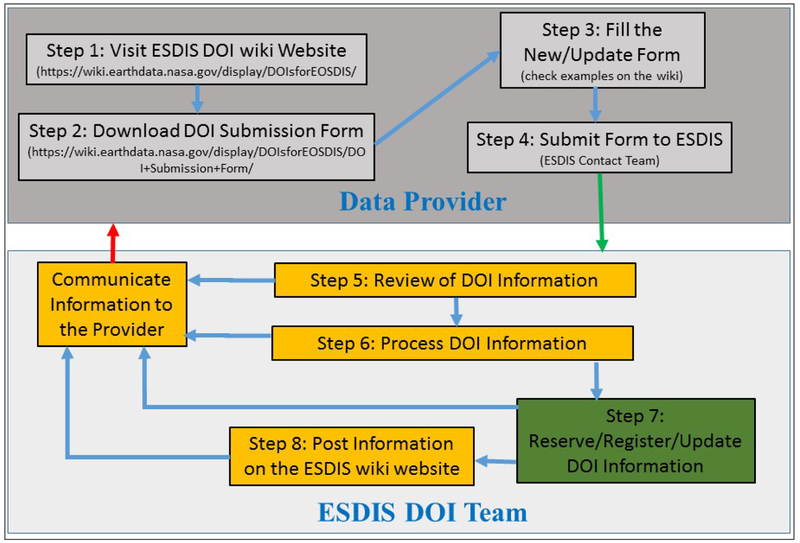
ESDIS Project DOI Process Workflow.

The DOI processing workflow as shown in Figure 1 is shared by the data provider and the ESDIS Project based on their respective responsibilities. The DOI process is initiated by the Data Provider’s submission of information to the ESDIS Project. Following this, the ESDIS Project staff reviews and validates the information as per the requirements. Based on the input provided by the DAACs, the system initiates the program to complete the process for reserving or registering. The outcome of the process is communicated to the data provider and, if required, data providers are informed of the action needed on their part.

## DOI Registration and Management System

**Figure 2:**
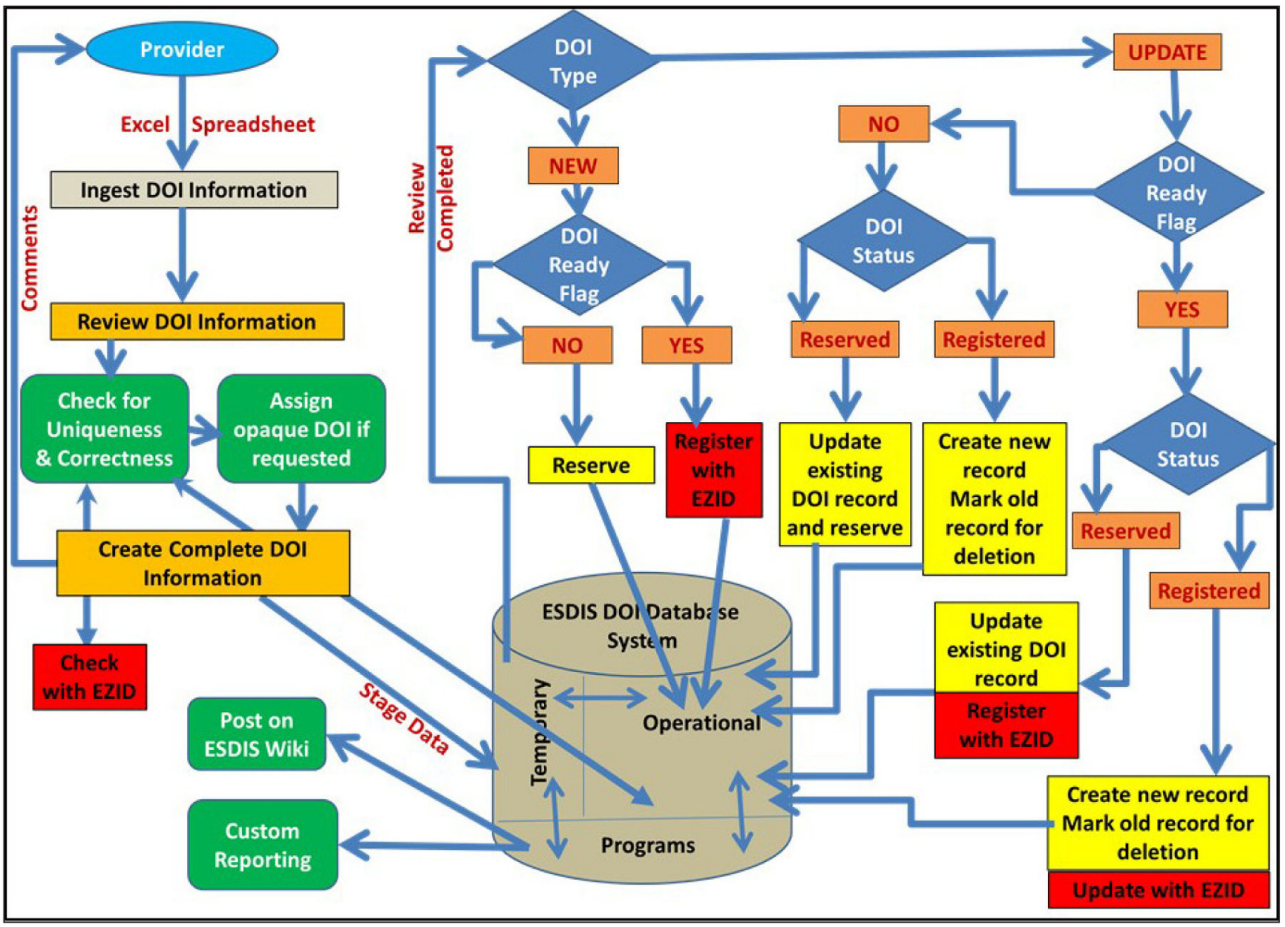
ESDIS Operational DOI Registration and Managing System.

DOI Registration and Management System is an information processing system for DOI name registration with EZID. It involves the collection of the DOI metadata as a data file, reviewing and validation of the information, and processing the data as shown in Figure 2. The system uses a tab-delimited data file derived from the excel spreadsheet that contains the metadata for the DOI provided by the data provider, programs written in PERL, and the Oracle database to store the DOI information.

Programs written in PERL were developed to perform automatic review and validation of metadata, and checking for DOI name uniqueness within the ESDIS database and with EZID. These programs also reserve, register, and update the DOI metadata within the ESDIS database and also with EZID. An Oracle database has been used to store all of the DOI information and some processing related metadata, such as submission date, registration date, and update date.

When a data provider is ready to register a DOI with EZID, complete processing of the DOI – starting with receiving the metadata attributes and ending with the registration as shown in Figure 2 – is performed, whether the DOI request is new or an update to the existing information. Also when data are being reviewed and validated the data are stored in a temporary table in the database. If there are any errors or additional information is required, the data provider is informed accordingly and the data are deleted from the temporary table. If all the attributes are correct and no action is required from data provider, the data are transferred to the permanent table in the database and processed to reserve, register, or update the DOI as per the request. If a request is to “Reserve” which is indicated by the value of “No” in the Ready for Registration attribute for the new DOI, the program performs review and validation but does not halt the process for incompleteness. As such, the information is archived in the ESDIS DOI database as “Reserve” until the data provider is ready to register. At that time, completeness of the required metadata attributes is enforced and the system will register the DOI with EZID only if all the mandatory metadata attributes are complete. The system also has the functionality of performing updates and deletions. Update processes are initiated based on the “Update” value in the type of request attribute and is applicable to the both “Reserve” and “Register” category. The deletion is applicable only to the “Reserve” (an unregistered) category. Once the information processing is completed, the DOI related information is posted on the unrestricted Earthdata wiki website (ESDIS 2016).

It is expected that the data products that have DOIs have support for the long-term management of the data collection, including archive and distribution. It has been established that DOIs can be assigned to each data collection and to the each version of the data collection. Occasionally, certain science data operations require that data products be either moved to another data center or deleted/deprecated, or replaced with a newer version. For the last four years, there has been no deprecation of the EOSDIS data products. Data centers have a process of deprecating data products based on practical resource considerations. Multiple versions of products may be maintained for smaller volume products, while older versions would be deleted for products whose volumes are large. For such large volume products, when the newer version of a product is released, the data centers continue distributing both versions for a brief period of overlap followed by the deletion of the older version. The following data management policy is being implemented regarding DOIs and landing pages. In all cases, when a new version of a product is created, a new DOI and landing page are created, but DOIs of the older versions are included in the new landing page. The older landing pages continue to be maintained, with pointers to the new landing page and a statement about the availability of the older version or lack thereof. In summary, the following are the new and unique features of the ESDIS DOI registration process:

- Data producer’s flexibility to reserve DOIs while the algorithm for the processing of the data products are being developed, which can be years before the product is actually made publicly available.
- The flexibility of assigning the data center that distributes the data product long after the DOI has been reserved.
- Easy transference of the responsibility of managing DOI and landing pages if and when the data products are transferred from one data center to another;
- An approach that assures registration of only one DOI for a data product that is distributed by two or more data centers by checking the DOI metadata during the registration process.
- The flexibility and option for the assignment of either opaque or structured DOIs based on the data centers’ requirements.
- A uniform DOI metadata system throughout EOSDIS that generates consistent citations in any specified format.
- Timely assessment of any changes in the DataCite Metadata Schema that can be analyzed with its impact by ESDIS and then the process can be revised accordingly without impacting EOSDIS data center resources and process.

## DOI Assignment Status

**Figure 3:**
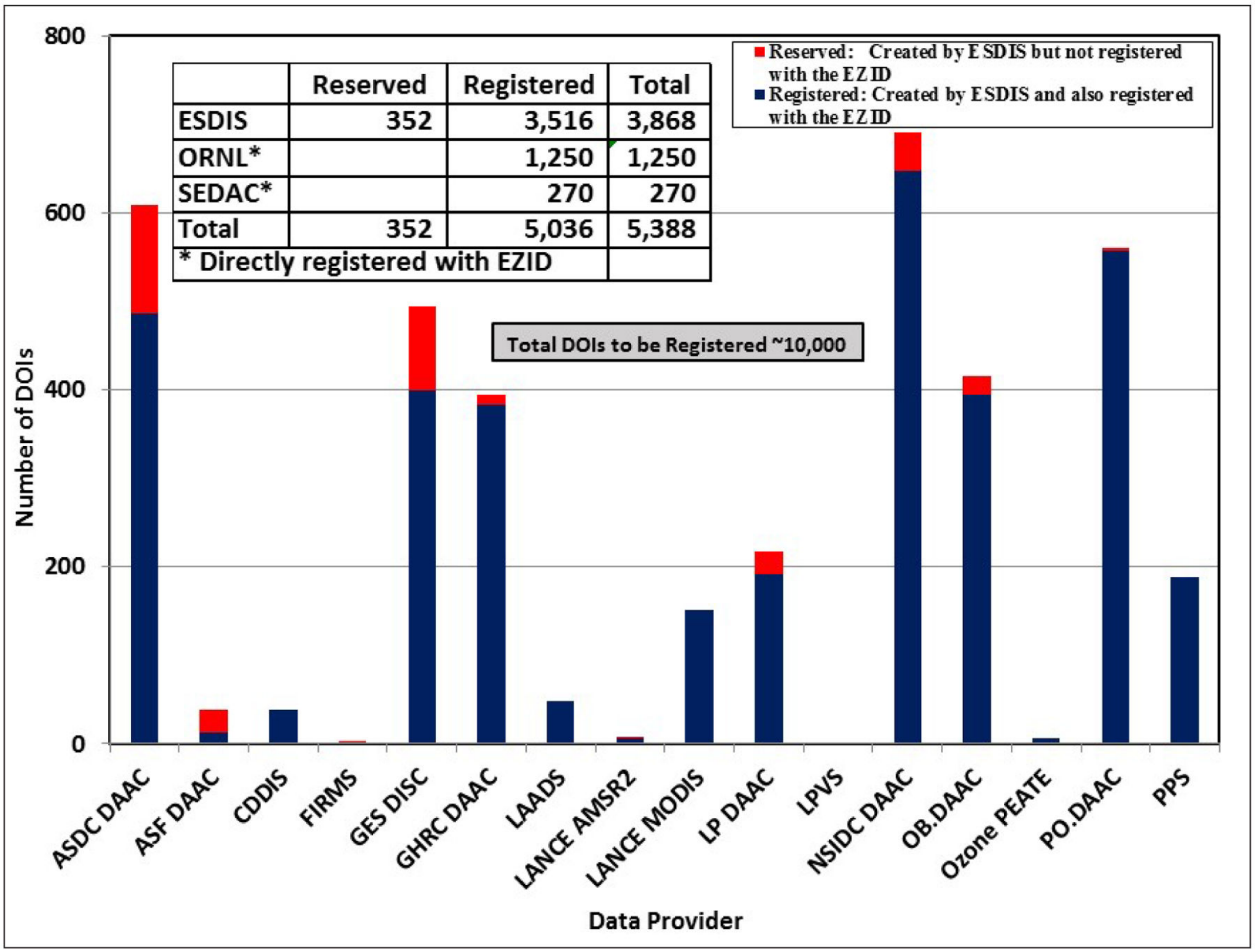
Status of ESDIS-created Digital Object Identifiers as of 28 February 2017.

Over the last five years, the ESDIS project has been assigning DOIs to products upon request by 10 of 12 DAACs and six non-DAAC organizations, four of which are closely associated with DAACs. These 16 organizations are shown in Table 2. The bar graph in Figure 3 shows the numbers of DOIs reserved and registered for each of these organizations as of 28 February 2017. In addition, the numbers of DOIs from the two DAACs (ORNL DAAC and SEDAC), which had been assigning DOIs to their data products before the ESDIS Project became a registration authority, are also shown in the summary table embedded in Figure 3.

The numbers in Figure 3 indicate that the about 50% of the products that are being currently distributed by all DAACs have registered DOIs. The automation implemented in the system has largely enabled the increase in the speed with which DOIs are being assigned. The variation in the number of DOIs among various data distributors is partly due to significant differences in numbers of data products managed by them. Also, given the relatively new ESDIS DOI process, it is more likely that data centers will choose to prioritize the assignment of DOIs to datasets recently received from active missions. Consequently, DAACs supporting a larger number of newer missions tend to show a higher number of registered DOIs.

The datasets that have not yet been assigned DOIs are primarily “legacy” data, i.e., old data collections that have been with the data centers for a very long time. Such data collections continue to be distributed, but developing DOIs for these products requires resources because these data collections’ metadata have to be re-written to include DOI-related information. The process also requires developing a landing page for each data collection. Once the information is included in the metadata, the data may have to be re-formatted and then archived. Data centers are requesting registration of DOIs for such products at a pace commensurate with the availability of resources.

## Discussion and Conclusion

In this paper, we have described the processes and an automated system that the ESDIS Project has been using for the last five years for assigning and registering DOIs to the data products held by the 12 EOSDIS DAACs and a few other related organizations. As of 28 February 2017, approximately 50% of the data products had registered DOIs. While initially the DOI assignment was slow, the automated process has resulted in significant increase in its speed.

**Figure 4:**
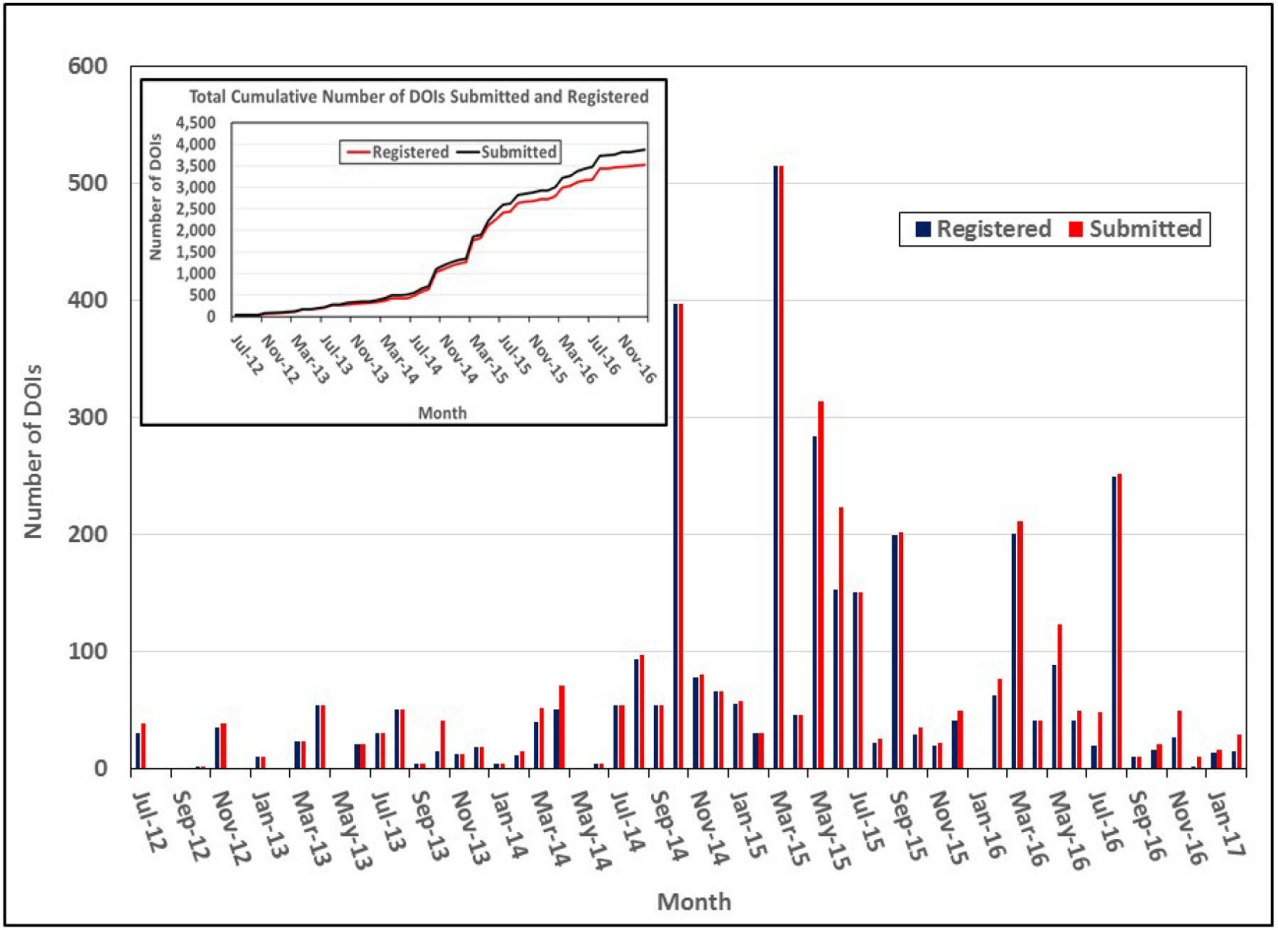
Monthly Number of DOI Requests Submitted by DAACS and Registered by the ESDIS DOI System as of February 2017.

In summary:

- DOI requests submitted by various DAACs vary from a single DOI to hundreds of DOIs that include requests for the assigning of opaque DOI names.
- The time period required for the processing has been an average of 1 to 2 seconds per DOI, which includes error checking, uniqueness verification, and registration with EZID. In addition, the process established has been able to identify some of the errors that could have compromised the DOI name registration, such as multiple back slashes, spaces between the letters in the suffix, and duplicate DOIs within the same file.
- This process has helped develop consistent naming conventions for various elements of the metadata across all data centers that are used in the development of the citation, such as the name of the data center that is distributing the data.
- The number of requests has been showing a steady monthly increase in recent years (Figure 4) compared to when the process was started by ESDIS;
- The system has demonstrated the capability of processing varied requests in a timely manner, and has provided the flexibility of reserving DOIs until the data provider is ready for DOIs to be resolved to landing pages;
- The gap between the cumulative numbers of the submitted and registered DOIs indicate that the DAACs do take advantage of the two-step process while they prepare the datasets for release and ensure that appropriate landing pages are created;
- The process ensures collection of high quality metadata attributes that are used in generating data citation;
- The ESDIS Project continues to provide policies and guidelines for the DOI process metadata requirements, and landing pages; and
- With this approach, the discoverability of the EOSDIS data products is increased and the data citation information will be easily accessible by the user community.

Given that the system allows opaque or structured DOI names, it would be interesting to observe their relative prevalence within the set of the ESDIS-registered DOIs. It has been observed that only 20% of these are opaque. A reason for data providers to request structured DOIs is that they may increase the ability of automated searches to identify articles that have cited specific mission or instrument or program datasets (or product-level) instead of actually reading through the articles to find which data have been referenced.

Currently, two policy decisions by the ESDIS Project are expected to result in registered DOIs for most of the EOSDIS data collections:

- One of the activities supported by the ESDIS Project, namely the Big Earth Data Initiative (BEDI), requires data centers to make selected data collections available to public users with certain defined services including having a registered DOI (Walter and McInerney 2016);
- The plan to make DOI registration a mandatory requirement for the metadata submitted to the Common Metadata Repository (CMR 2017) that is used for searching EOSDIS data products by users.

In addition, data citations are becoming more common, and more publishers are mandating data citations that include DOIs (COPDESS 2015). Various publishers encourage citation of datasets that include a persistent method for identification that is machine-actionable, globally unique, and widely used by a community (DCSG 2014). ESDIS DOI registration has been in place for the last five years, and it will take some time for citations of datasets using the DOIs to grow in scientific literature. To examine the extent of their use, a few Google Scholar searches were performed to collect information on the articles that cite “10.5067” and “NASA” in the references or the text of articles. The referenced DOIs appeared in refereed journal articles, books, and posters. After excluding articles that seemed not to be relevant for this analysis, there were over 370 articles in 2016 that cite DOIs compared to approximately 100 in 2015. These counts do not account for multiple DOIs referenced in one article. This shows a significant increase in the usage of ESDIS-registered DOIs in the citations. Such usage is expected to show a multifold increase as additional data products are being assigned DOIs and publishers are requiring authors to provide data citations.

## Figures and Tables

**Table 1:** List of DOI Metadata Attributes.

Metadata Attribute	Description
***Mandatory:***	
**DOI Name**	DOI name based on the defined syntax (Leave blank if requesting opaque IDs); required by EZID
**Product Title**	Title of the Product; required by EZID
**Creator**	Person(s)/Agency that has developed/processed the data; required by EZID
**Distributor**	Name of the agency that is distributing the data; required by EZID
**Year**	Year the data (will be) made available to public; required by EZID
**Product URL**	Landing Page URL; required by EZID
**Resource Type**	Type of the Digital Object, such as Dataset, Text, Services, Software; required by EZID
**Type of DOI (NEW or UPDATE)**	NEW for providing the DOI information for the first time or UPDATE for revising already provided information. Required by the ESDIS Project for processing of information
**Ready for Registration With EZID (YES or NO)**	Specifies if the Submitter is ready for registering the identifier with the EZID. This requires complete metadata and that the landing page should be ready: YES for Ready to Register, NO for Not ready to Register. Required by the ESDIS Project for processing of information
***Optional (ESDIS Project Internal Use):***	
**Special Reference**	Data Center preferred reference of the data product (short name)
**Program/Mission Name**	Name of the Program (e.g., MEaSUREs) or Mission (e.g., Aqua) for reference
**Project/Instrument Name**	Name of the Project (e.g., GSSTF) or Instrument (e.g., MODIS) for reference
**DOI Information Provider**	Name of the Data Center requesting the DOI assignment.
**DOI Contact Information**	First Name, Last Name, and Email address of the person providing the information
**Landing Page Responsible Agency**	Organization maintaining the landing page if it is different from the Data Center
**Landing Page Contact Information**	First Name, Last Name, and Email address of the person responsible for the landing page

**Table 2:** Name and Science Discipline of Various Data Distributors.

Data Distributor	Data Distributor Name	Science Discipline
**ASDC DAAC**	Langley Atmospheric Science Data Center DAAC	Radiation Budget, Clouds, Aerosol, Tropospheric Chemistry
**ASF DAAC**	Alaska Satellite Facility DAAC	Synthetic Aperture Radar Products, Sea Ice, Polar Processes, Geophysics.
**CDDIS**	Crustal Dynamics Data Information System	Space Geodesy, Solid Earth
**FIRMS**^1^	Land Atmosphere Near real-time Capability for EOS Fire Information for Resource Management System	Near real-time active fire data
**GES DISC**	Goddard Earth Sciences Data and Information Services Center	Global Precipitation, Solar Irradiance, Atmospheric Composition and Dynamics, Global Modeling
**GHRC DAAC**	Global Hydrology Resource Center DAAC	Hydrologic Cycle, Severe Weather Interactions, Lightening, Atmospheric Convention
**LAADS**	Level 1 and Atmosphere Archive and Distribution System	MODIS Level-1 and Atmospheric Products
**LANCE AMSR2**^1^	Land Atmosphere Near-real-time Capability for EOS (LANCE) AMSR2 at the GHRC DAAC	Near real-time global precipitation and ocean parameters excluding sea surface temperature.
**LANCE MODIS**^1^	LANCE MODIS at the MODAPS	Near real-time Land and Atmospheric data
**LPDAAC**	Land Processes DAAC	Surface Reflectance, Land Cover, Vegetation Indices
**LPVS**^2^	Land Product Validation Subgroup (Working Group on Calibration and Validation Committee on Earth Observation Satellites)	Land Product Validation
**NSIDC DAAC**	National Snow and Ice Data Center DAAC	Snow and Ice, Cryosphere, Climate Interactions, Sea Ice
**OB.DAAC**	Ocean Biology DAAC	Ocean Biology, Sea Surface Temperature
**Ozone PEATE**^1^	Ozone Product Evaluation and Test Element	Ozone Measurements
**ORNL**	Oak Ridge National Laboratory DAAC	Biogeochemcial Dynamics Ecological Data, Environmental Processes
**PO.DAAC**	Physical Oceanography DAAC	Gravity, Sea Surface Temperature, Ocean Winds, Topography, Circulation and Currents
**PPS**^1^	Precipitation Processing System	Global Precipitation Measurement
**SEDAC**	Socioeconomic Data and Application Data Center	Human Interactions, Land Use, Environmental Sustainability, Geospatial Data

1 Non-DAAC organization associated with a DAAC.

2 Non-DAAC organization.

## References

[R1] CMR 2017 Common Metadata Repository (CMR). Available at: https://earthdata.nasa.gov/about/science-system-description/eosdis-components/common-metadata-repository (Last accessed on Feb 7, 2017).

[R2] COPDESS 2015 COPDESS statement of commitment. Available at: http://www.copdess.org/statement-of-commitment/ (Last accessed 28 February 2017).

[R3] DataCite Metadata Working Group 2016 DataCite Metadata Schema for the Publication and Citation of Research Data v4.0, DataCite e.V (Last accessed October 28, 2016). DOI: 10.5438/0012

[R4] DCSG 2014 Data Citation Synthesis Group: Joint Declaration of Data Citation Principles. In: Martone M (Ed.) San Diego CA: FORCE11; 2014. Available at: https://www.force11.org/group/joint-declaration-data-citation-principles-final.

[R5] Duerr RE, Downs RR, Tilmes C, 2011 On the utility of identification schemes for digital Earth science data: An assessment and recommendations. Earth Science Informatics, 4: 139 DOI: 10.1007/s12145-011-0083-6

[R6] ESDIS 2016 Digital Object Identifiers (DOI) for EOSDIS, Earth Science Data and Information System (ESDIS) GSFC/NASA Greenbelt, MD, USA Available at: https://wiki.earthdata.nasa.gov/display/DOIsforEOSDIS/ (Last accessed February 27, 2017).

[R7] ESIP 2012 Data Citation Guidelines for Data Providers and Archives, The Federation of Earth Science Information Partners (ESIP). Available at: http://commons.esipfed.org/node/308 (Last accessed on Feb 7, 2017).DOI: 10.7269/P34F1NNJ.

[R8] Moses J and Behnke J 2012 Digital Object Identifiers for NASA’s Earth Observing System. The Earth Observer, 24(5): 10–15. Available at: https://eospso.gsfc.nasa.gov/sites/default/files/eo_pdfs/Sept_Oct_2012_color_508.pdf.

[R9] Murphy K and Ramapriyan H 2016 Collaborations and Partnerships in NASA’s Earth Science Data Systems Program SciDataCon 2016. Denver, Colorado: USA Available at: http://www.scidatacon.org/2016/sessions/35/paper/87/.

[R10] Walter J and McInerney M 2016 Evolving the Management and Dissemination of NASA Earth Observation Data in a Big Data World, OGC Location Powers Workshop – Orlando, FL, Presented on September 20, 2016 Available at: https://portal.opengeospatial.org/files/70965.

[R11] Wanchoo L and James N 2014 ESDIS DOI System, Approach, and Future Direction, Poster presented at American Geophysical Union, AGU Paper Number: IN13B-1570, Dec 2014

